# *Pseudomonas aeruginosa* Reduces VX-809 Stimulated F508del-CFTR Chloride Secretion by Airway Epithelial Cells

**DOI:** 10.1371/journal.pone.0127742

**Published:** 2015-05-27

**Authors:** Bruce A. Stanton, Bonita Coutermarsh, Roxanna Barnaby, Deborah Hogan

**Affiliations:** Department of Microbiology and Immunology, The Geisel School of Medicine at Dartmouth, Hanover, New Hampshire, United States of America; University of Alabama at Birmingham, UNITED STATES

## Abstract

**Background:**

*P*. *aeruginosa* is an opportunistic pathogen that chronically infects the lungs of 85% of adult patients with Cystic Fibrosis (CF). Previously, we demonstrated that *P*. *aeruginosa* reduced wt-CFTR Cl secretion by airway epithelial cells. Recently, a new investigational drug VX-809 has been shown to increase F508del-CFTR Cl secretion in human bronchial epithelial (HBE) cells, and, in combination with VX-770, to increase FEV1 (forced expiratory volume in 1 second) by an average of 3-5% in CF patients homozygous for the F508del-CFTR mutation. We propose that *P*. *aeruginosa* infection of CF lungs reduces VX-809 + VX-770- stimulated F508del-CFTR Cl secretion, and thereby reduces the clinical efficacy of VX-809 + VX-770.

**Methods and Results:**

F508del-CFBE cells and primary cultures of CF-HBE cells (F508del/F508del) were exposed to VX-809 alone or a combination of VX-809 + VX-770 for 48 hours and the effect of *P*. *aeruginosa* on F508del-CFTR Cl secretion was measured in Ussing chambers. The effect of VX-809 on F508del-CFTR abundance was measured by cell surface biotinylation and western blot analysis. PAO1, PA14, PAK and 6 clinical isolates of *P*. *aeruginosa* (3 mucoid and 3 non-mucoid) significantly reduced drug stimulated F508del-CFTR Cl secretion, and plasma membrane F508del-CFTR.

**Conclusion:**

The observation that *P*. *aeruginosa* reduces VX-809 and VX-809 + VX-770 stimulated F508del CFTR Cl secretion may explain, in part, why VX-809 + VX-770 has modest efficacy in clinical trials.

## Introduction

CFTR is a cyclic-AMP regulated Cl channel localized to the apical plasma membrane of epithelial cells in the lungs [1–4]. Cl secretion via wt-CFTR is the major driving force for the production of a thin layer of liquid overlying the lung epithelium, which is essential for effective mucociliary transport that mechanically clears debris and pathogens from the airways and, thereby, serves a vital role in innate immunity [4–6]. Mutations in the *CFTR* gene cause Cystic Fibrosis (CF), an autosomal recessive genetic disease that causes progressive loss of lung function and death in the 3^rd^ decade of life due to a decrease in airway surface liquid and reduced mucociliary transport, leading to chronic bacterial lung infections [1–3,6]. The F508del mutation in CFTR increases its degradation in the endoplasmic reticulum, dramatically reducing CFTR mediated Cl secretion [7,8]. In addition, the F508del mutation reduces the half-life of CFTR and the single channel open probability by ~50% [9,10]. Recently, Vertex Pharmaceuticals developed VX-809 (Lumacaftor), which increases the amount of F508del-CFTR in the plasma membrane of airway epithelial cells, and VX-770 (Ivacaftor), which increases the open probability of F508del-CFTR, to be given together to CF patients homozygous for the F508del CFTR mutation [9,11,12]. Together these drugs increase F508del-CFTR Cl secretion by human bronchial epithelia cells in Ussing chamber experiments to a level predicted to improve lung function in CF patients. Clinical trials with a combination of VX-809 + VX-770 have been promising, with an overall modest improvement in FEV1 of ~3–5% [11].

Previously, we demonstrated that *P*. *aeruginosa* reduces wt-CFTR Cl secretion by airway epithelial cells by a mechanism mediated in part by the secretion of Cif (CFTR inhibitory factor), a virulence factor present in outer membrane vesicles, which enhances the ubiquitination and degradation of wt-CFTR [12–14]. Thus, we propose that *P*. *aeruginosa* infection of the CF lungs, which is apparent in ~85% of adult CF patients, reduces VX-809 stimulated F508del-CFTR Cl secretion, thereby reducing the efficacy of VX-809 + VX-770. Accordingly, the goal of this study was to test the hypothesis that *P*. *aeruginosa* reduces VX-809 stimulated F508del-CFTR Cl secretion in human CF airway epithelial cells. We report that *P*. *aeruginosa* reduced VX-809, and VX809 + VX-770 stimulated Cl secretion in a CF cell line (CFBE cells) and in CF primary cultures of human bronchial epithelial (HBE) cells homozygous for F508del-CFTR. Furthermore, the effects were observed in all nine *P*. *aeruginosa* isolates tested, including those with the alginate-overproducing mucoid phenotype that is common among strains from long-term CF infections. Because ~85% of adult CF patients are chronically colonized by *P*. *aeruginosa*, these observations may explain, in part, why VX-809 + VX-770 has only modest effects on FEV1 in CF patients with the F508del/F508del-CFTR mutation.

## Materials and Methods

### Cell culture

Primary CF human bronchial epithelial cells (hereafter referred to as CF-HBE cells) were obtained from the University of North Carolina courtesy of Dr. Scott Randell, and maintained in culture as described [15]. The Dartmouth Committee for the Protection of Human Subjects has determined that the use of CF-HBE cells in this study is not considered human subject's research because cells are taken from discarded tissue and contain no patient identifiers. CF-HBE cells secrete mucus and have apical cilia. Cells from a minimum of three donors with the F508del/F508del mutation were used in all studies. CFBE41o- cells, homozygous for the F508del mutation, and stably expressing F508del-CFTR (hereafter referred to as CFBE cells) were generously provided by Dr. J.P. Clancy, University of Cincinnati. CFBE cells were studied between passages 18 and 27, and grown in culture as described in detail [16,17]. Briefly, to establish confluent, polarized monolayers, 0.5× 10^6^ CFBE cells were seeded onto 24-mm Transwell permeable supports or 12mm Snapwell permeable supports (0.4μm pore size, Corning, Corning, NY) coated with Vitrogen plating medium containing human fibronectin (10 μg/ml, Collaborative Biomedical Products, Bedford, MA), PureCol (1%, Advanced BioMatrix, San Diego, CA), and bovine serum albumin (10 μg/ml, Invitrogen) and grown in an air-liquid interface culture at 37°C for 6–9 days, as described [18,19]. To establish confluent, polarized monolayers of CF-HBE cells, 1 × 10^6^ cells were seeded onto 24-mm Transwell permeable supports or 250,000 cells were seeded onto 12mm Snapwell permeable supports (0.4-μm pore size, Corning, Corning, NY) coated with 50μg/ml Collagen type IV (Sigma) and grown in an air-liquid interface culture at 37°C for 3–4 weeks, as described [15]. Cells grown on Transwell filters were used for biochemical studies and cytokine analysis, and cells grown on Snapwell filters were used in Ussing chamber experiments.

### Bacterial strains and growth conditions

In these studies, we used *P*. *aeruginosa* strains PAO1, PA14 and PAK, and six clinical isolates of *P*. *aeruginosa* (three mucoid: SMC1585, SMC5450, SMC5451 and three non mucoid: SMC1587, SMC1595, SMC1596) isolated from the sputa of six independent CF patients at the Dartmouth—Hitchcock Medical Center (Hanover, NH, USA). In addition, studies were conducted with *Staphylococcus newman* and *Streptococcus salavari*. All *P*. *aeruginosa* strains and *Streptococcus salavari* were grown and maintained in LB medium (Lysogeny Broth, LB) at 37°C [20]. *Staphylococcus newman* was grown in THY broth with Oxyrase. For co-culture studies, *P*. *aeruginosa*, *Staphylococcus newman* or *Streptococcus salavari* were harvested from overnight cultures, washed twice in CFBE cell-growth medium, and then suspended in cell-growth medium without antibiotics or phenol red. The cell suspensions were added in 300 μl of cell growth medium to the apical face of CFBE or CF-HBE monolayers for 6 hours. For control monolayers the same volume of fluid, without bacteria, was added to the apical face of CFBE and CF-HBE cells. None of the *P*. *aeruginosa* isolates or *Staphylococcus newman* and *Streptococcus salavari* had any effect on LDH release by CFBE cells over the course of the experiment (n = 3/group), indicating that the bacteria studied had no effect on epithelial cell viability.

### Ussing chamber analysis of F508del-CFTR Cl secretion

Ussing chamber measurements of F508del-CFTR Cl secretion were performed as described [16,17]. Briefly, CFBE and CF-HBE cells grown on Snapwell permeable supports were mounted in an Ussing chamber (Physiologic Instruments, San Diego, CA) and short circuit current (*I*_sc_) was measured by voltage-clamping the transepithelial voltage across the monolayers to 0 mV with a voltage clamp (Physiologic Instruments). Amiloride (50 μM) was added to the apical bath solution (5 ml total volume in the apical and basolateral bath solutions) to inhibit *I*_sc_ attributed to sodium reabsorption, and subsequently *I*_sc_ was stimulated with forskolin (10 μm), followed by VX-770 (5 μm) to stimulate CFTR mediated *I*_sc_, and thiazolidinone (CFTR_inh_-172, 20 μm; EMD Millipore, Billerica, MA) to inhibit CFTR-mediated *I*_sc_. Data are expressed as CFTR_inh_-172 inhibited *I*_sc_ in μA/cm^2^. Data collection and analysis were done with the Acquire & Analyze Data Acquisition System (Physiologic Instruments). To examine the effect of *P*. *aeruginosa* on F508del-CFTR Cl secretion *P*. *aeruginosa* (see above for strains) was added to the apical side of CFBE and CF-HBE cell monolayers, which have a layer of mucus overlying cells, at a multiplicity of infection (MOI) of 30:1 for 6 hours in the absence of antibiotics, and then *I*_sc_ was measured as described above. Ussing chamber studies were also conducted to examine the effect of *Staphylococcus newman and Streptococcus salavari* on VX-809 stimulated F508del-CFTR Cl secretion by CFBE cells, as described above for *P*. *aeruginosa*.

### Biochemical determination of apical membrane CFTR and transferrin receptor

Briefly, the biochemical determination of F508del-CFTR in the apical plasma membrane of cells was performed by domain selective cell surface biotinylation using EZ-Link Sulfo-NHS-LC-Biotin (Pierce) at 4°C, as described previously in detail [18,23]. PAO1 was added to the apical side of CFBE monolayers at a multiplicity of infection (MOI) of 30:1 for 6 hours in the absence of antibiotics, and, subsequently, biotinylated proteins were isolated by streptavidin-agarose beads, eluted into SDS sample buffer, and separated by 7.5% SDS-PAGE. The blots were probed for F508del-CFTR, Transferrin receptor (TfR) and for Na^+^-K^+^-ATPase as a loading control. Western blot analysis of F508del-CFTR, TfR and Na^+^-K^+^-ATPase were conducted by methods described previously [16,21].

### Antibodies and reagents

The antibodies used were: mouse monoclonal anti-CFTR antibody (clone 596; University of North Carolina Cystic Fibrosis Center, Chapel Hill, NC) 1:1000 dilution; mouse monoclonal anti-TfR (0.5mg/ml, catalog #13–6800, Invitrogen, Camarillo, CA) 1:1000 dilution; mouse monoclonal anti-Na^+^-K^+^-ATPase (DSHB Hybridoma Product a5 developed by D.M. Fambrough, Department of Biology, The Johns Hopkins University) was obtained from the Developmental Studies Hybridoma Bank, created by the NICHD of the NIH and maintained at The University of Iowa, Department of Biology, Iowa City, IA 52242.; goat anti-mouse IgG ((H + L)-HRP Conjugate; BioRad catalog #170–6516) 1:3000 dilution; and goat anti-rabbit IgG ((H + L)-HRP Conjugate BioRad catalog #170–6515, Bio-Rad, Hercules, CA) 1:3000 dilution.

### Cytokines

IL-6 and IL-8 produced by polarized monolayers of CFBE and CF-HBE cells were measured using the Bio-Rad Bio-Plex cytokine arrays (Hercules, CA) as described [19,24]. CFBE and CF-HBE cells were grown on Transwell filters as described above, and *P*. *aeruginosa* was added to the apical side of monolayers at a multiplicity of infection (MOI) of 30:1 for 1 h in the absence of antibiotics, and subsequently *P*. *aeruginosa* was removed by replacing the apical medium with MEM. Five hours after *P*. *aeruginosa* was washed from monolayers the apical and basolateral media were removed for analysis of cytokines.

### Data analysis and statistics

Graphpad Prism version 5.0 for Macintosh (Graphpad, San Diego, CA) was used to perform a statistical analysis of the data. Means were compared using a t-test or ANOVA followed by Tukeys test, as appropriate. P<0.05 was significant, and all data are expressed as the mean ± SEM.

## Results

### VX-809 stimulates F508del-CFTR Cl secretion

**Fig 1:**
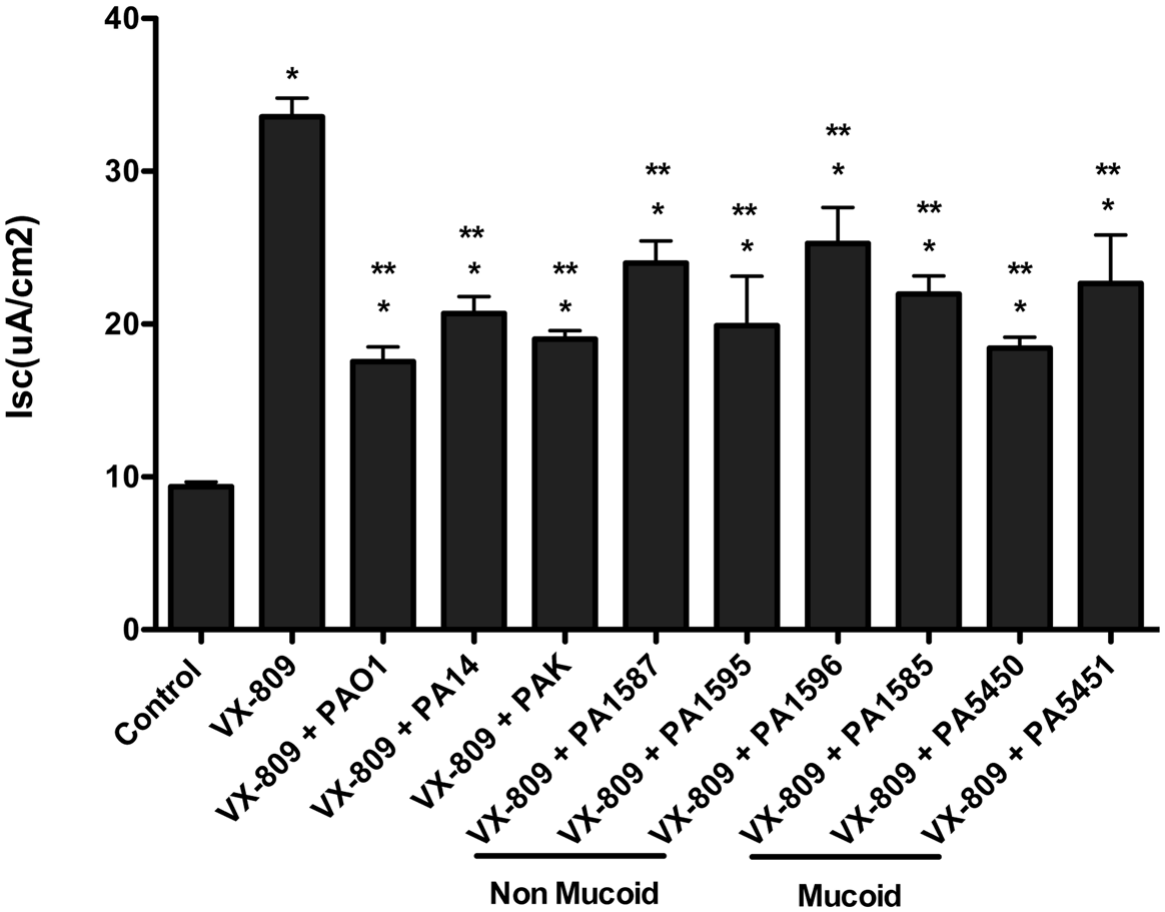
A. Effects of VX-809 alone and in combination with *P*. *aeruginosa* on CFBE cells. VX-809 (3 μM, 48 hours) increased Cl secretion compared to vehicle treated control in CFBE cells. PA01, PA14, PAK and 6 clinical isolates of *P*. *aeruginosa* reduced VX-809-stimulated F508del-CFTR Cl secretion compared to VX-809 alone. *P<0.05 versus control. **P<0.05 versus VX-809. N = 3 to 46/treatment.

**Fig 2:**
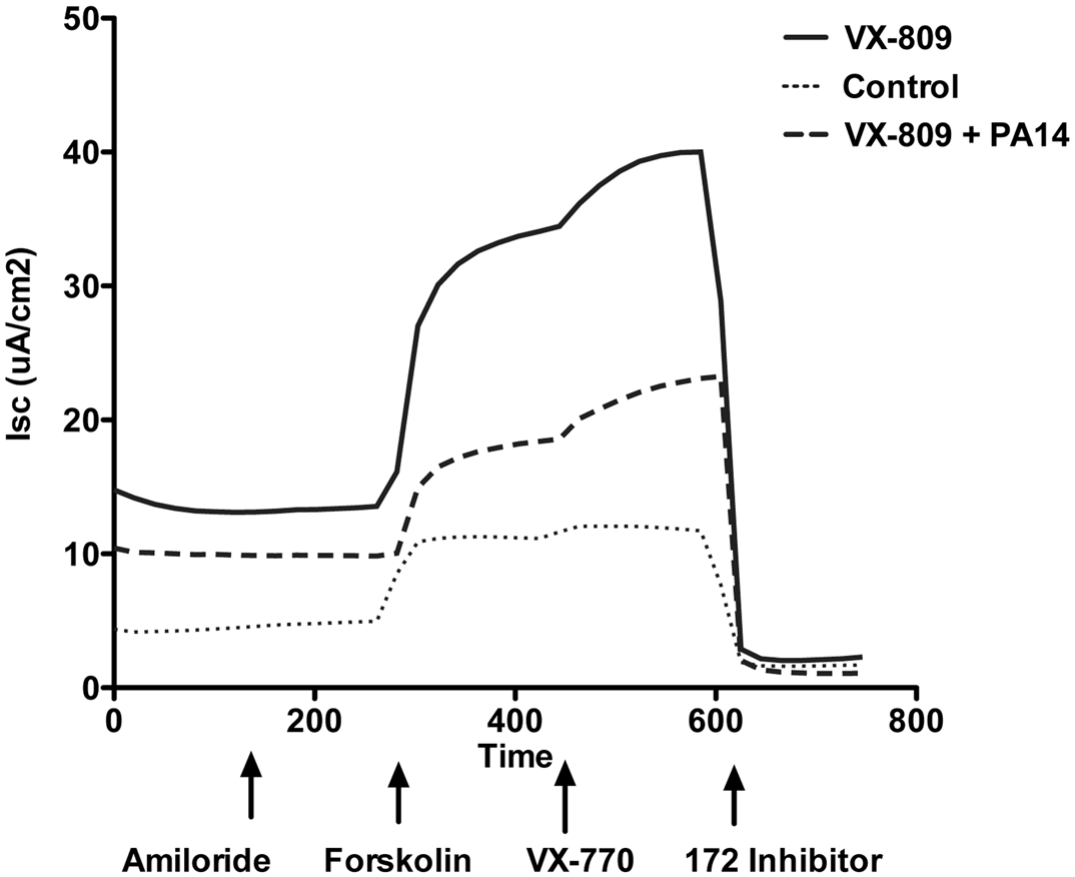
Representative current traces of CFBE cells. Amiloride (50 μM) was added to the apical bath solution to inhibit the short circuit current (*I*_sc_) attributed to sodium reabsorption, and subsequently *I*_sc_ was stimulated with forskolin (FK, 10 μm), followed by VX-770 (5 μm), and thiazolidinone (CFTR_inh_-172, 20 μm; EMD Millipore, Billerica, MA) to inhibit CFTR-mediated *I*_sc_.

**Fig 3:**
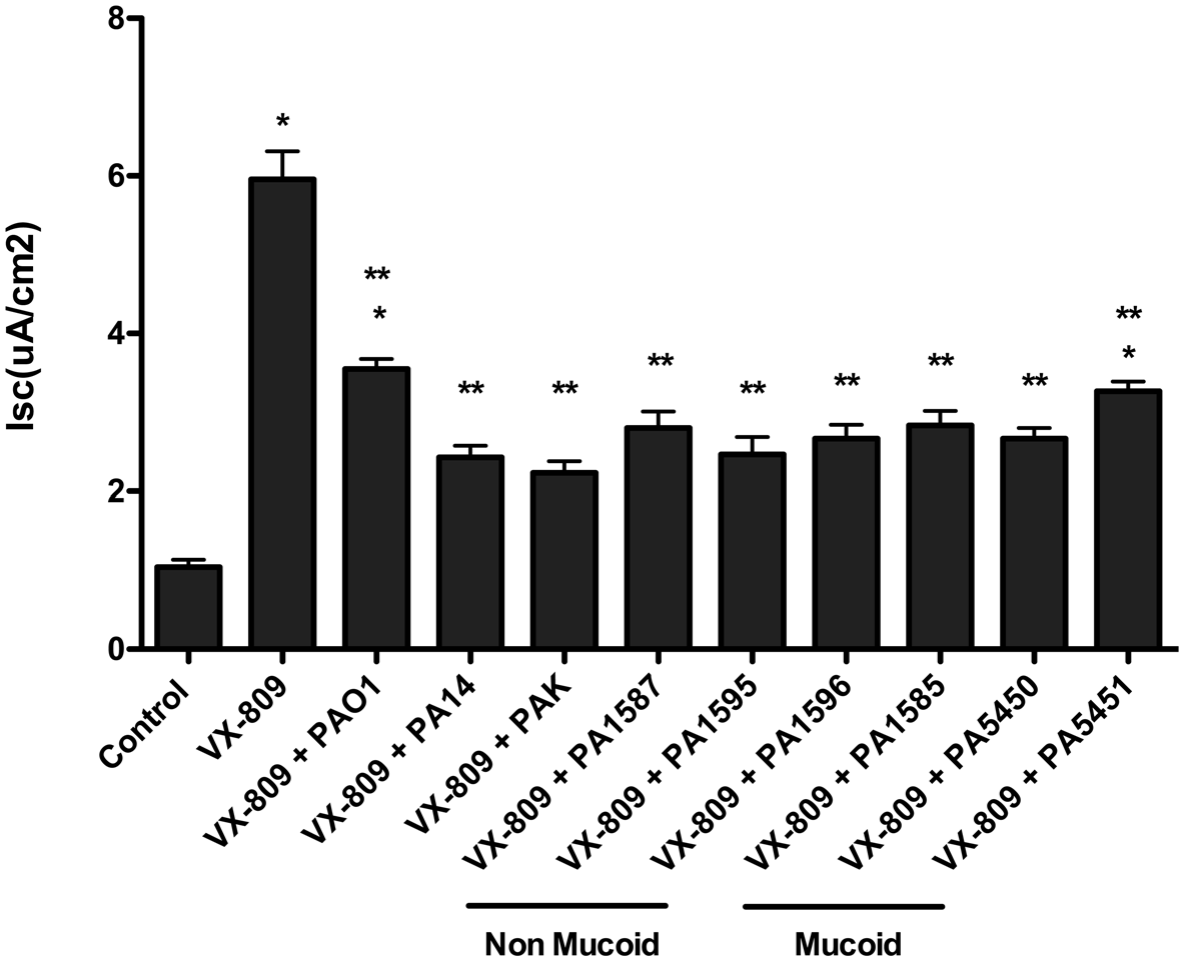
Effects of VX-809 alone and in combination with *P*. *aeruginosa* on primary CF-HBE cells. VX-809 (3 μM, 48 hours.) increased Cl secretion compared to vehicle treated control in primary cultures of CF-HBE cells. PA01, PA14, PAK and 6 clinical isolates of *P*. *aeruginosa* reduced VX-809-stimulated F508del-CFTR Cl secretion compared to VX-809 alone. *P<0.05 versus control. **P<0.05 versus VX-809. N = 3 donors, from each donor 3 to 19 monolayers of cells were studied/treatment.

VX-809 (3 μM for 48 hours) increased F508del-CFTR Cl secretion by CFBE cells (Fig 1, S1 Table). Three laboratory isolates of *P*. *aeruginosa* (PAO1, PA14 and PAK) reduced VX-809-stimulated F508del-CFTR Cl secretion by 48%, 38% and 43%, respectively, after 6 hours of co-incubation (See Fig 2, S2 Table for representative traces of F508del-CFTR Cl currents). Additional studies were conducted using recent clinical isolates of *P*. *aeruginosa*, including three non-mucoid and three mucoid strains obtained from CF patients at the Dartmouth Hitchcock Medical Center [20]. All of the clinical isolates caused a significant reduction in VX-809 stimulated F508del-CFTR Cl secretion in CFBE cells, with a range of 25% to 45% inhibition (Fig 1, S1 Table). Additional studies were performed to determine if *P*. *aeruginosa* also impacted drug rescued F508del-CFTR Cl secretion in CF-HBE cells in primary culture. As seen in CFBE cells, PAO1, PA14 and PAK reduced the VX-809-stimulated F508del-CFTR Cl secretion (41%, 60% and 63%, respectively) (Fig 3, S3 Table). Moreover, three non-mucoid and three mucoid strains also reduced VX-809 stimulated F508del-CFTR Cl secretion in CF-HBE cells, with a range of 46% to 59% inhibition (Fig 3, S3 Table).

**Fig 4:**
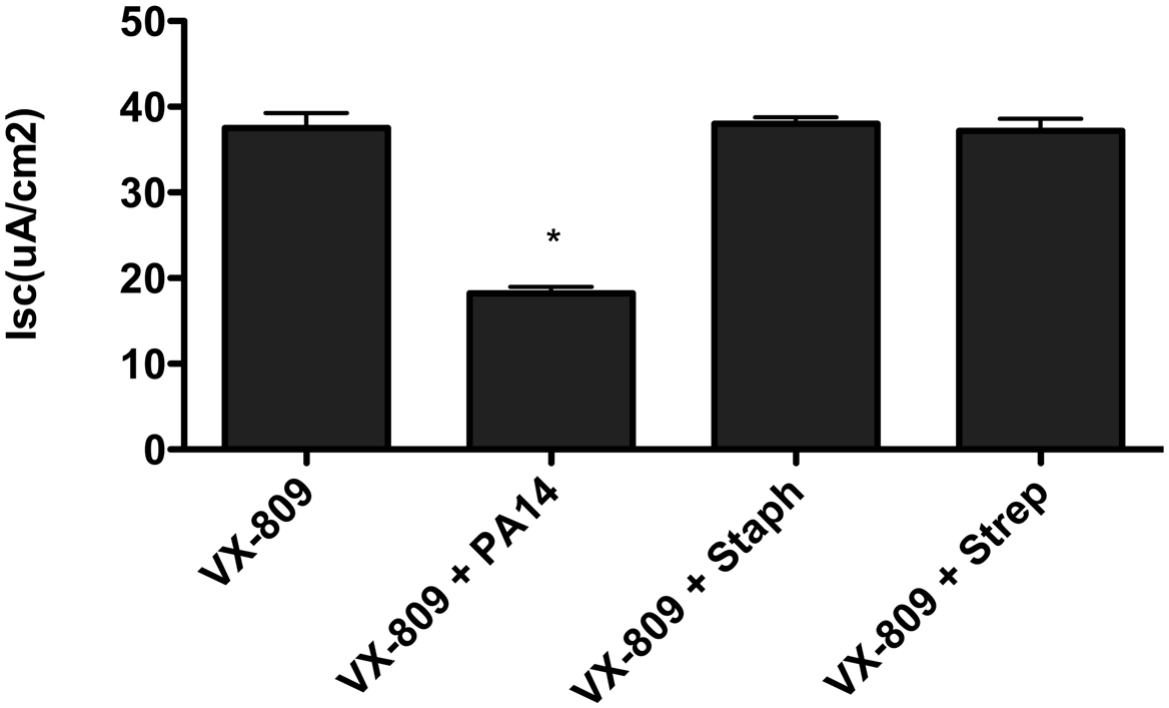
PA14 reduced VX-809 (3 μM, 48 hours.) stimulated Cl secretion by CFBE cells. Neither *Staphylococcus newman* (Staph) nor *Streptococcus salivari* (Strep) reduced VX-809-stimulated F508del-CFTR Cl secretion compared to VX-809 alone. *P<0.01 versus VX-809, Strep and Staph. N = 3/monolayers/group.

Although *P*. *aeruginosa* reduced VX-809 stimulated F508del-CFTR Cl secretion in CFBE cells and in CF-HBE cells in primary culture, neither *Staphylococcus newman* nor *Streptococcus salavari* had any effect on VX-809 stimulated F508del-CFTR Cl secretion in CFBE cells (Fig 4, S4 Table).

**Fig 5:**
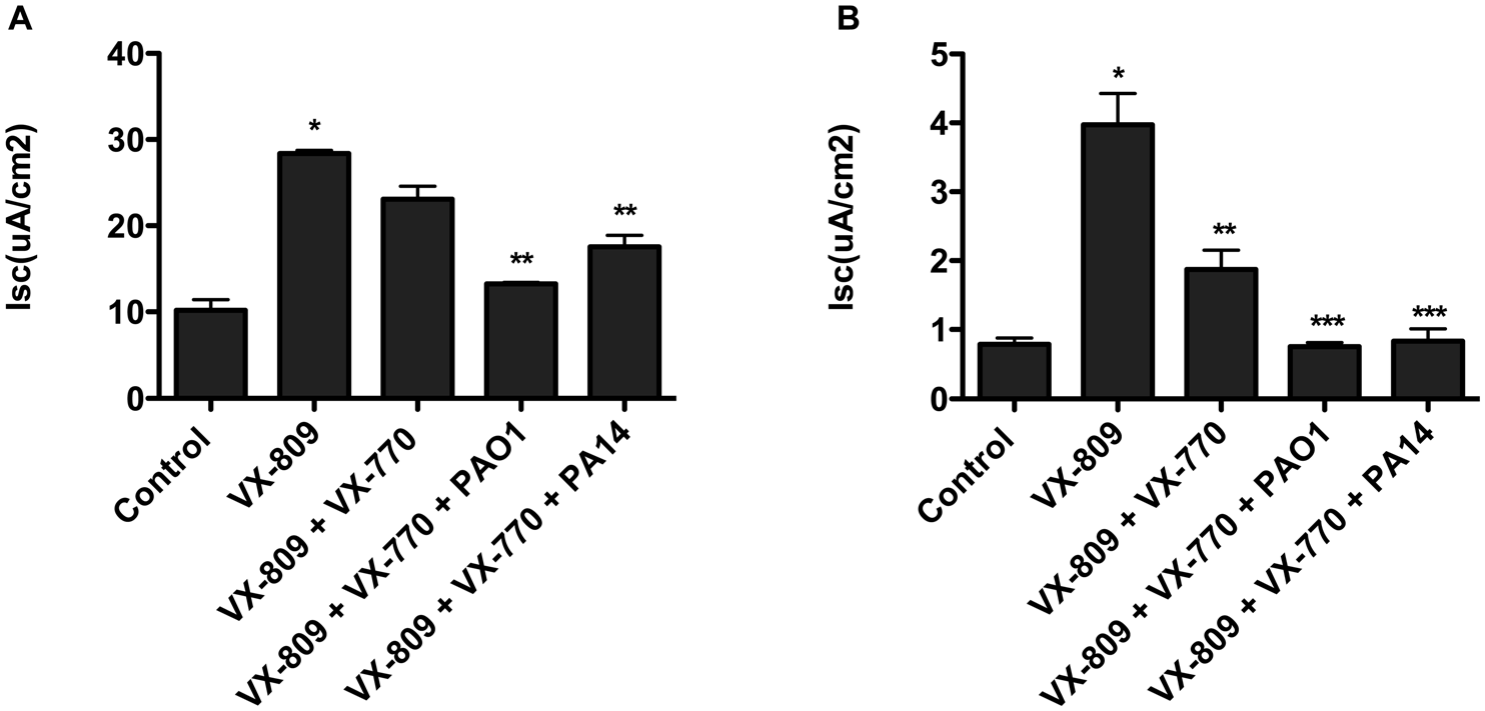
Effect of *P*. *aeruginosa* on F508del-CFTR Cl secretion in CFBE cells (A) and CF-HBE cells (B) treated with VX-809 alone and VX-809 + VX-770. VX-770 (5 μM) did not significantly alter VX-809 (3 μM) stimulated Cl secretion in CFBE cells. Both PAO1 and PA14 reduced VX-809 + VX-770 stimulated Cl secretion in CFBE cells. By contrast VX-770 significantly reduced VX-809 stimulated Cl secretion in CF-HBE cells. As in CFBE cells both PAO1 and PA14 reduced the VX-809 + VX-770 stimulated Cl secretion. *P<0.05 versus control. **P<0.05 versus VX-809. ***P<0.05 versus VX-809 + VX-770. N = 3 to 6/treatment for CFBE cells. For CF-HBE cells, N = 3 donors, from each donor 3 to 7 monolayers of cells were studied/treatment.

Recently, as the studies in this report were being written for publication, two groups independently reported that co-administration of VX-770 with VX-809 for 48 hours decreased the ability of VX-809 to increase F508del-CFTR Cl currents in primary cultures of CF-HBE cells [22,23]. Thus, we conducted additional experiments with the co-administration of VX-809 + VX-770 (48 hours) to examine the effect of *P*. *aeruginosa* on F508del-CFTR Cl secretion. Fig 5, S5 Table demonstrates that VX-809 (3 μM) alone and in combination with VX-770 (5 μM) for 48 hours stimulated F508del-CFTR Cl secretion, although Cl secretion was less in CF-HBE cells treated with VX809 + VX-770 compared with cells treated for 48 hrs. with VX-809 alone. Interestingly, F508del-CFTR Cl secretion was not significantly reduced by VX-770 in CFBE cells treated with VX-809. However, in both CFBE cells and in CF-HBE cells treated with VX-809 + VX-770, *P*. *aeruginosa* (PAO1 and PA14) reduced F508del-CFTR Cl secretion (Fig 5, S5 Table).

### *P*. *aeruginosa* reduces F508del-CFTR in the plasma membrane

**Fig 6:**
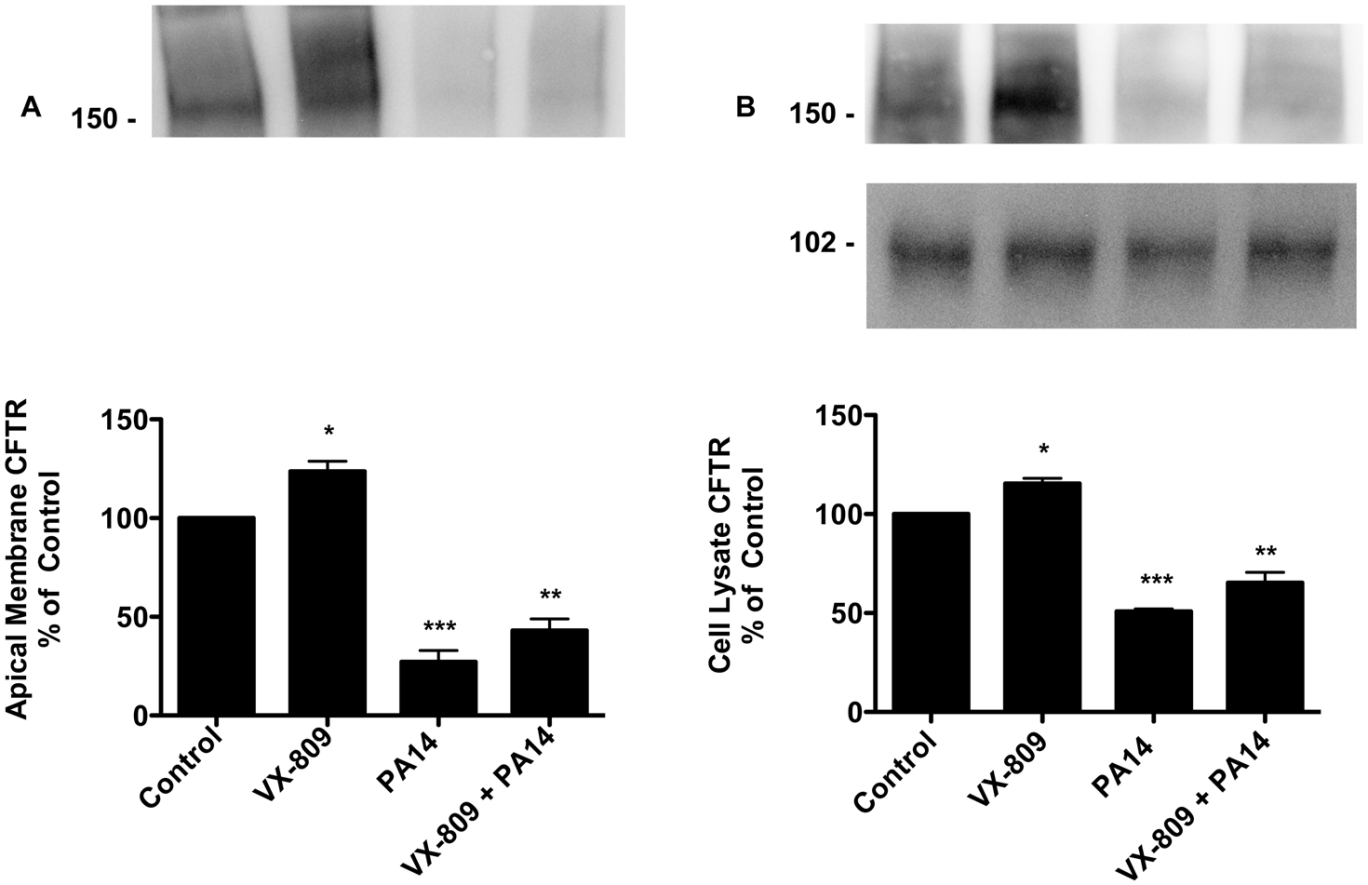
Analysis of apical CFTR in the presence of VX-809 alone and in combination with *P*. *aeruginosa*. (A) Western blot of apical membrane F508del-CFTR in CFBE cells treated with vehicle (Control), VX-809 (3 μM) alone, PA14 alone, or VX-809 (3 μM) + PA14. Na^+^-K^+^-ATPase is a loading control. *P<0.05 versus all other groups. N = 4/group. **P<0.05 versus VX-809 alone, ***P<0.05 versus Control. N = 4/group. (B) Top: Representative western blot of F508del-CFTR in cell lysates of CFBE cells treated with vehicle (Control), VX-809 (3 μM) alone, PA14 alone, or VX-809 (3 μM) + PA14. Bottom: Representative western blot of Na^+^-K^+^-ATPase (gel loading control) in cell lysates of CFBE cells treated with vehicle (Control), VX-809 (3 μM) alone, PA14 alone, or VX-809 (3 μM) + PA14 *P<0.05 versus all other groups. **P<0.05 versus VX-809 alone, ***P<0.05 versus Control. N = 4/group. All samples run on the same gel but cut for presentation. N = 4/treatment.

**Fig 7:**
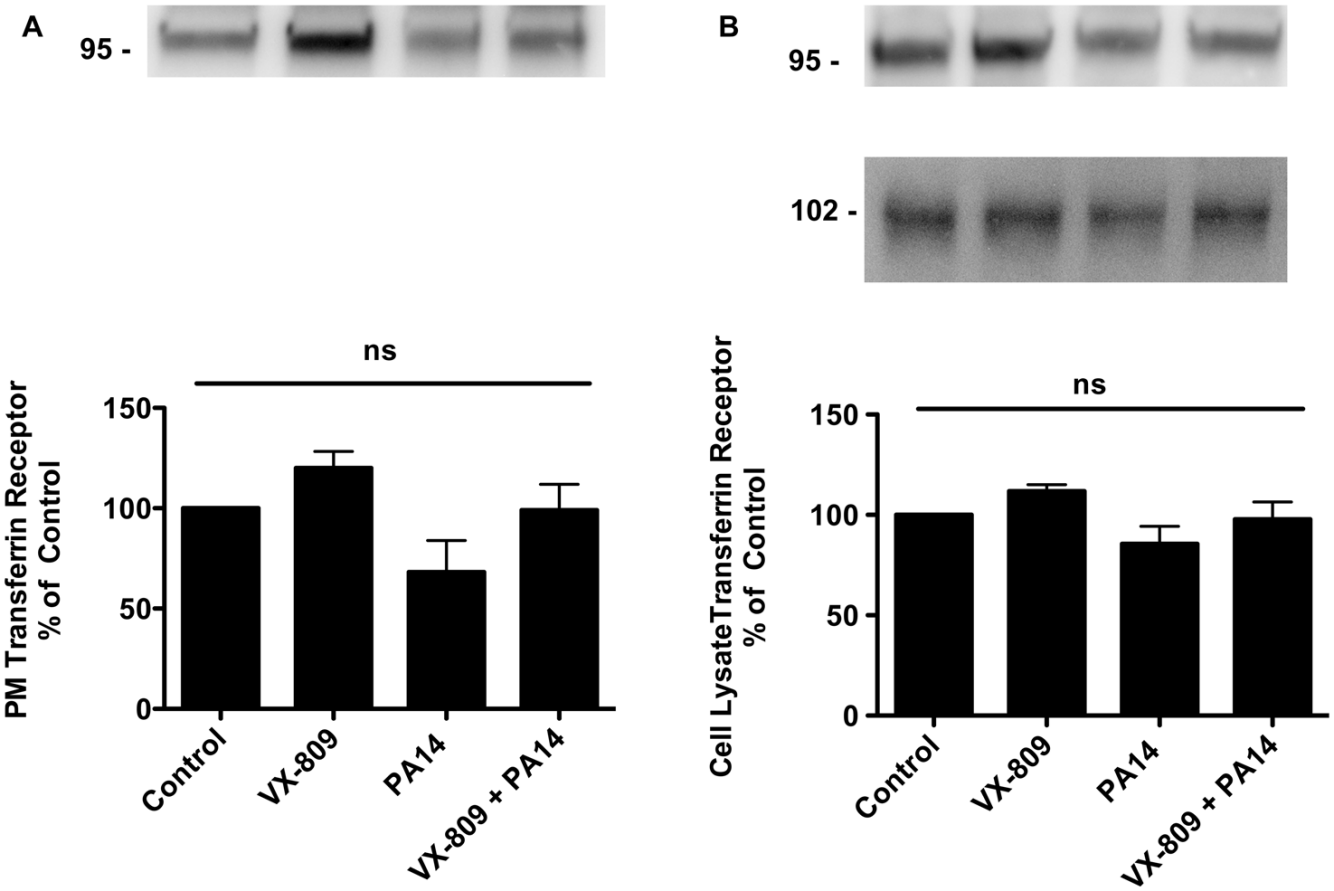
Analysis of plasma membrane transferrin receptor in the presence of VX-809 alone and in combination with *P*. *aeruginosa*. (A) Representative western blot of apical plasma membrane transferrin receptor (TfR) in CFBE cells treated with vehicle (Control), VX-809 (3 μM), PA14 alone, or VX-809 (3 μM) + PA14. ns, not significantly different. N = 3/treatment. (B) Top: Representative western blot of cell lysate TfR in CFBE cells treated with vehicle (Control), VX-809 (3 μM), PA14 alone, or VX-809 (3 μM) + PA14. Bottom: Representative western blot of cell lysate Na^+^-K^+^-ATPase in CFBE cells treated with vehicle (Control), VX-809 (3 μM), PA14 alone, or VX-809 (3 μM) + PA14. N = 4/treatment. ns, not significantly different. All samples run on the same gel but cut for presentation.

Previous studies have shown that VX-809 reduces the degradation of F508del-CFTR in the proteasome, and increases plasma membrane F508del-CFTR [24]. Thus, studies were conducted to determine if *P*. *aeruginosa* reduces VX-809 stimulated F508del-CFTR Cl secretion by reducing F508del-CFTR in the apical cell membrane of CFBE cells. VX-809 increased the amount of F508del-CFTR in the apical membrane of CFBE cells as well as in cell lysates (Fig 6, S1, S2 and S4 Figs). However, *P*. *aeruginosa* reversed the VX-809-stimulated increase in F508del-CFTR in both the plasma membrane and cell lysate (Fig 6, S1, S2 and S4 Figs). By contrast, neither VX-809 nor *P*. *aeruginosa* had any effect on the amount of transferrin receptor in the plasma membrane or in cell lysates of CFBE cells (Fig 7, S3 and S4 Figs). Thus, *P*. *aeruginosa* reduced VX-809 stimulated F508-CFTR Cl secretion, in part, by decreasing the amount of F508del-CFTR in the apical plasma membrane.

### VX-809 has no effect on IL-6 and IL-8 secretion

**Fig 8:**
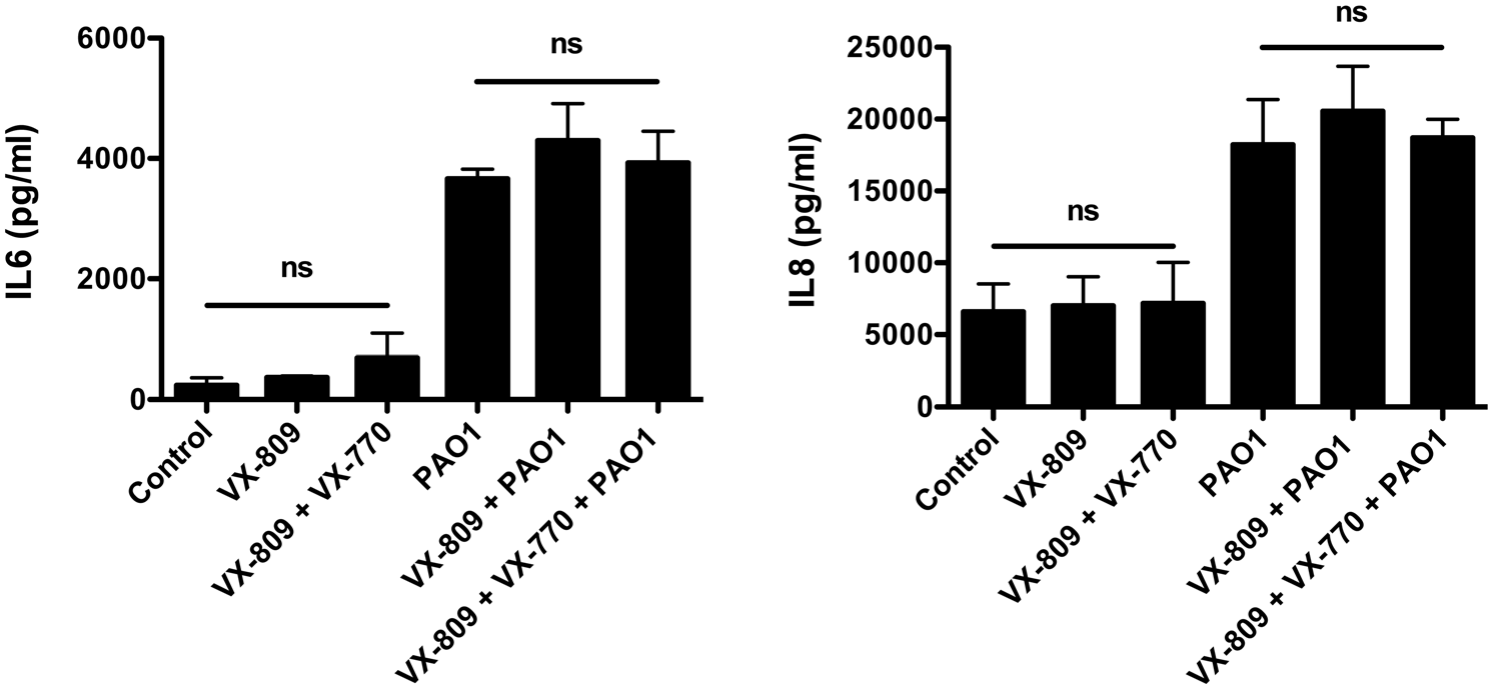
Analysis of cytokines production in response to *P*. *aeruginosa* in the presence and absence of CFTR modulating compounds. VX-809 (3 μM) alone and VX-809 + VX-770 (5 μM) had no effect on constitutive or PAO1 stimulated IL-6 and IL-8 secretion by CFBE cells (A) or CF-HBE cells (B). All PAO1 treated P<0.05 versus untreated. N = 3-6/treatment.

In a previous study, we reported that neither VX-325 nor Corr4a, investigational compounds that increase F505del-CFTR Cl secretion, had any effect on cytokine secretion by CFBE cells treated with vehicle or *P*. *aeruginosa* [17]. Thus, studies were conducted to determine if VX-809 alone or VX-809 + VX-770 reduced cytokine secretion by CFBE and CF-HBE cells. Neither VX-809 alone nor VX-809 + VX-770 had a significant effect on constitutive IL-6 and IL-8 secretion, or on PAO1 simulated IL-6 and IL-8 secretion in CFBE or CF-HBE cells (Fig 8).

## Discussion

This study demonstrates that 3 well characterized laboratory strains and 6 clinical isolates of *P*. *aeruginosa* (3 mucoid and 3 non-mucoid) inhibit the VX-809 and VX-809 + VX-770 stimulated increase in F508del-CFTR Cl secretion in a cell line (CFBE) and in primary cultures of CF-HBE cells. The *P*. *aeruginosa* induced inhibition of VX-809 stimulated F508del-CFTR Cl secretion is similar to that reported previously for the effect of *P*. *aeruginosa* on wt-CFTR Cl secretion [14,16,27]. The observation that *P*. *aeruginosa* reduces VX-809 + VX-770 stimulated F508del-CFTR Cl secretion, coupled with the fact that ~85% of adult CF patients are colonized with *P*. *aeruginosa* suggests that the modest effect of VX-809 + VX-770 on FEV1 in CF patients (3–5% increase) may be due in part to chronic infection with *P*. *aeruginosa*.

If *P*. *aeruginosa* reduces VX-809 + VX-770 stimulated F508del-CFTR Cl secretion *in vivo* then two predictions can be made. First, if *P*. *aeruginosa* inhibits VX-809 + VX-770 stimulated F508del-CFTR Cl secretion one would expect to see a disproportional improvement in sweat [Cl] compared to the improvement in FEV1, since sweat ducts are not infected with *P*. *aeruginosa*. Recent clinical trials with VX-809 + VX-770 contain several examples where there is a disproportional improvement in sweat [Cl] in subjects treated with VX-809 + VX-770 [25]. For example, in Cohort #1 VX-809 + VX-770 (VX-809, 200 mg once per day + VX-770, 250 mg every 12h) decreased sweat [Cl] from day 1 to day 21 by 12.6 mmol/L, a 12.6% decrease but had no significant effect on FEV1. For Cohort #2, VX-809 + VX-770 (VX-809, 400 mg once per day + VX-770, 250 mg every 12h) decreased sweat [Cl] from day 1 to day 56 by 9.1 mmol/L, a 9.1% decrease, and increased FEV1 by 3.6% [25]. Thus, these two examples reveal a discordance between changes in sweat [Cl] and FEV1. Boyle et al. [25] have suggested that the differential effects of VX-809 + VX-770 on sweat [Cl] and FEV1 may be related to different tissue distribution of VX-809/VX-770, differences in the biology of CFTR in sweat duct and lungs, or non-CFTR effects of VX-809/VX-770. We suggest that another reason is that *P*. *aeruginosa*, which infects the lungs but not sweat ducts, inhibits VX-809 + VX-770 stimulated F508del-CFTR Cl secretion.

In addition, if *P*. *aeruginosa* inhibits VX-809 + VX-770 stimulated F508del-CFTR Cl secretion one would expect to see a disproportional improvement in FEV1 in subjects not infected with *P*. *aeruginosa* compared to those who are infected with *P*. *aeruginosa*. However, at the North American CF Conference in 2014 data were presented demonstrating that there was no significant difference between the effect of VX-809 + VX-770 on FEV1 in subjects positive or negative for *P*. *aeruginosa* [11]. Although this observation is at odds with our conclusion that infection with *P*. *aeruginosa* may reduce the efficacy of VX-809 + VX-770 *in vivo*, it is important to note that other bacteria in the CF lungs, including *Acinetobacter nosocomialis* and *Acinetobacter baumannii* secrete a virulence factor (aCif) that reduces CFTR abundance in CFBE cells [26], thus, it is possible that infection with other bacterial that are known to reduce CFTR abundance, in addition to *P*. *aeruginosa*, may influence the response to VX-809 + VX-770. Clearly, additional studies are required to determine if *P*. *aeruginosa*, and or infection with other bacteria such as *Acinetobacter* that reduces CFTR abundance, suppresses the efficacy of VX-809 + VX-770 *in vivo*.

In two recent studies, it was reported that VX-770 dramatically reduced the ability of VX-809 to increase F508del-CFTR Cl secretion [22,23]. This is at odds with clinical trials with VX-809 + VX-770 that demonstrated significant improvement in FEV1 and several other improvements in clinical outcomes in CF patients homozygous for the F508del-CFTR mutation [11], as well as the studies in this report in which the combination of VX-809 + VX770 (48 hours) significantly increased F508del-CFTR Cl secretion in CFBE cells and in CF-HBE cells (Figs 1–3 and 5, S1–S3 and S5 Tables). Several factors may contribute to the variable effects of VX-809 + VX-770 on F508del-CFTR Cl secretion by HBE cells, including the possible differential expression of modifier genes among donors studied that may affect export of F508del-CFTR from the endoplasmic reticulum, endocytic trafficking of F508del-CFTR and channel open probability [27,28]. While additional experiments are required to determine why these studies reach different conclusions regarding the effect of VX-770 on VX-809 stimulated F508del-CFTR Cl secretion, our data show that *P*. *aeruginosa* inhibits both the VX-809 and the VX-809 + VX-770 stimulated increase in F508del-CFTR Cl secretion in primary cultures of CF-HBE cells.

We have shown that several laboratory strains of *P*. *aeruginosa* (PAO1, PA14 and PAK) as well as 6 clinical isolates of *P*. *aeruginosa* reduce VX-809 (and VX-809 + VX-770) stimulated F508del-CFTR Cl secretion. Furthermore, preliminary studies presented by Guimbellot and colleagues at the 2014 North American Cystic Fibrosis Conference confirm our results that *P*. *aeruginosa* reduces VX-809 stimulated F508del-CFTR Cl secretion [29]. Interestingly, these authors demonstrated that other bacteria known to colonize the CF lung, including *Haemophilus influenza* and *Staphylococcus aureus*, had no effect on F508del-CFTR Cl secretion. Our results with *Staphylococcus newman* and *Streptococcus salivari* demonstrate that these bacteria also have no effect on VX-809 stimulated F508del-CFTR Cl secretion.

Finally, studies were also conducted to determine if VX-809 alone or VX-809 + VX-770 reduced *P*. *aeruginosa* induce cytokine production. A reduction of cytokine secretion would be beneficial in CF patients since the hyperinflammatory lung milieu that has been linked to worse clinical outcomes in CF [30]. However, neither VX-809 alone nor VX-809 + VX-770 reduced constitutive nor the *P*. *aeruginosa* stimulated increase in IL-6 and IL-8 secretion by CFBE or CF-HBE cells, a result consistent with a previous study in which we demonstrated that two other “correctors”, VX-325 and Corr 4a, had no effect on the *P*. *aeruginosa* induced inflammatory response by CFBE cells [17]. Taken together these studies from our laboratory and from others demonstrate that although VX-325, VX-809 and Corr4a increase F508del-CFTR Cl secretion, these drugs do not modify the hyperinflammatory response of CF airway cells to *P*. *aeruginosa* infection. The most effective drugs for CF patients would ideally enhance F508del-CFTR Cl secretion and reduce the proinflamatory response to bacterial infection.

## Conclusion

Because 85% of adult CF patients are chronically colonized by *P*. *aeruginosa*, and because *P*. *aeruginosa* inhibited VX-809 and VX-809 +VX-770 F508del-CFTR Cl secretion by CFBE and CF-HBE cells, the observation in this paper may explain, in part, why VX-809 + VX-770 has only modest clinical benefit.

## Supporting Information

S1 Fig Western blots of apical membrane CFTR used to prepare Fig 6A. Vertical line indicates where blot was cut for presentation.
(TIF)

S2 Fig Western blots of CFTR in cell lysates used to prepare Fig 6B. Vertical line indicates where blot was cut for presentation purposes. Band near 76 kDa standard was from reprobe with a non-CFTR antibody.
(TIF)

S3 Fig Western blots of cell lysate and plasma membrane transferrin receptor expression used in Fig 7A (plasma membrane) and 7B (cell lysate). Vertical lines indicate where blot was cut for presentation purposes. Band in top blots near 102 kDa was from a reprobe with a commercial Na/K/ATPase antibody that did not recognize Na/K/ATPase.
(TIF)

S4 Fig Na/K/ATPase blots probed with antibody from Developmental Studies Hybridoma Bank used to normalize CFTR and transferrin to prepare Figs 6 and 7. Vertical lines indicate where blot was cut for presentation purposes.
(TIF)

S1 Table Raw data from Fig 1. (XLSX)

S2 Table Raw data from Fig 2. (XLSX)

S3 Table Raw data from Fig 3. (XLSX)

S4 Table Raw data from Fig 4. (XLSX)

S5 Table Raw data from Fig 5. (XLSX)
