# Supplementary figures and images for: Pseudomonas aeruginosa Reduces VX-809 Stimulated F508del-CFTR Chloride Secretion by Airway Epithelial Cells
Source: PLoS One. 2015 May 27;10(5):e0127742. doi: 10.1371/journal.pone.0127742 (PMC4446214; doi:10.1371/journal.pone.0127742)

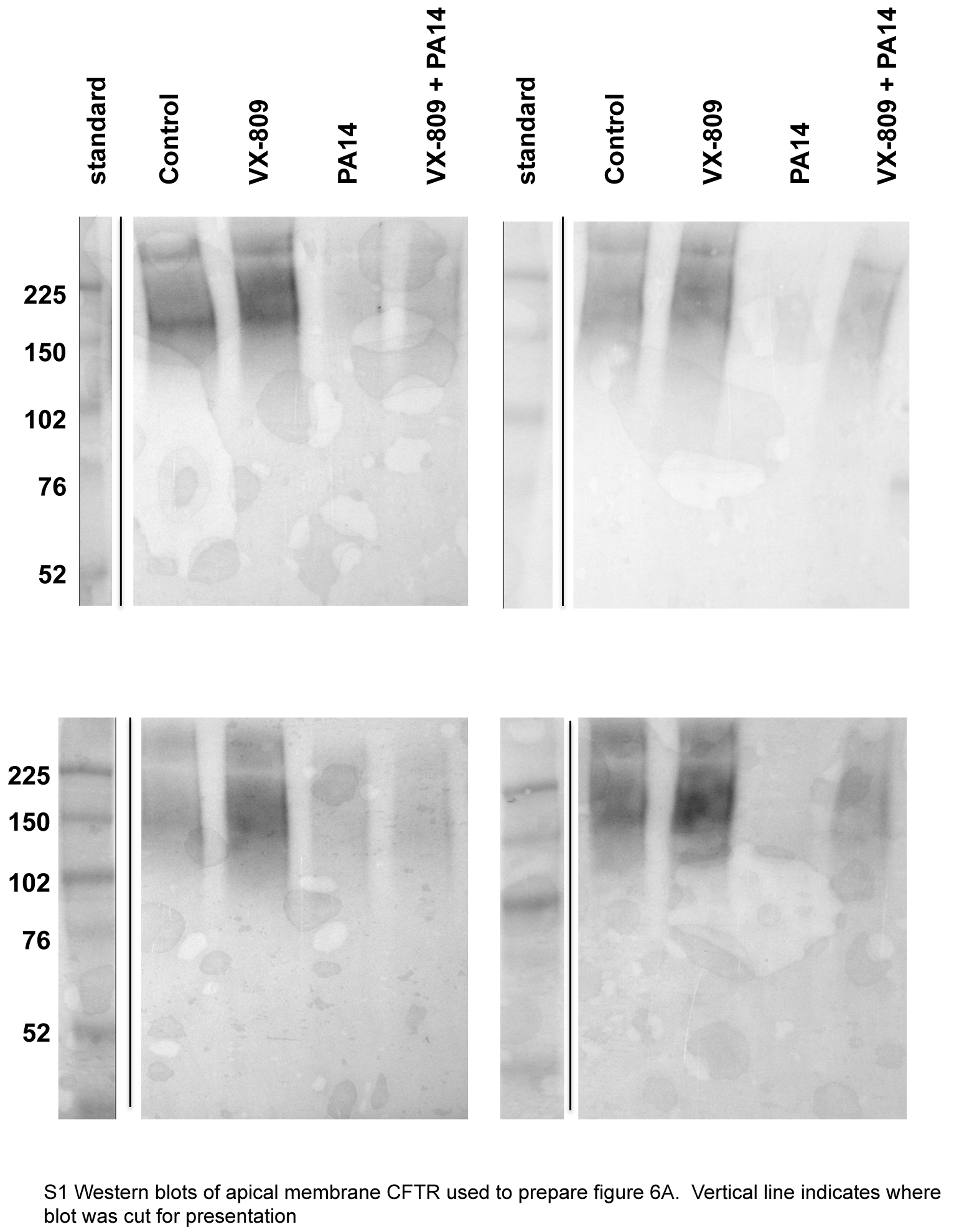

Supplement: S1 Fig — Vertical line indicates where blot was cut for presentation. (TIF) [file pone.0127742.s001.tif]

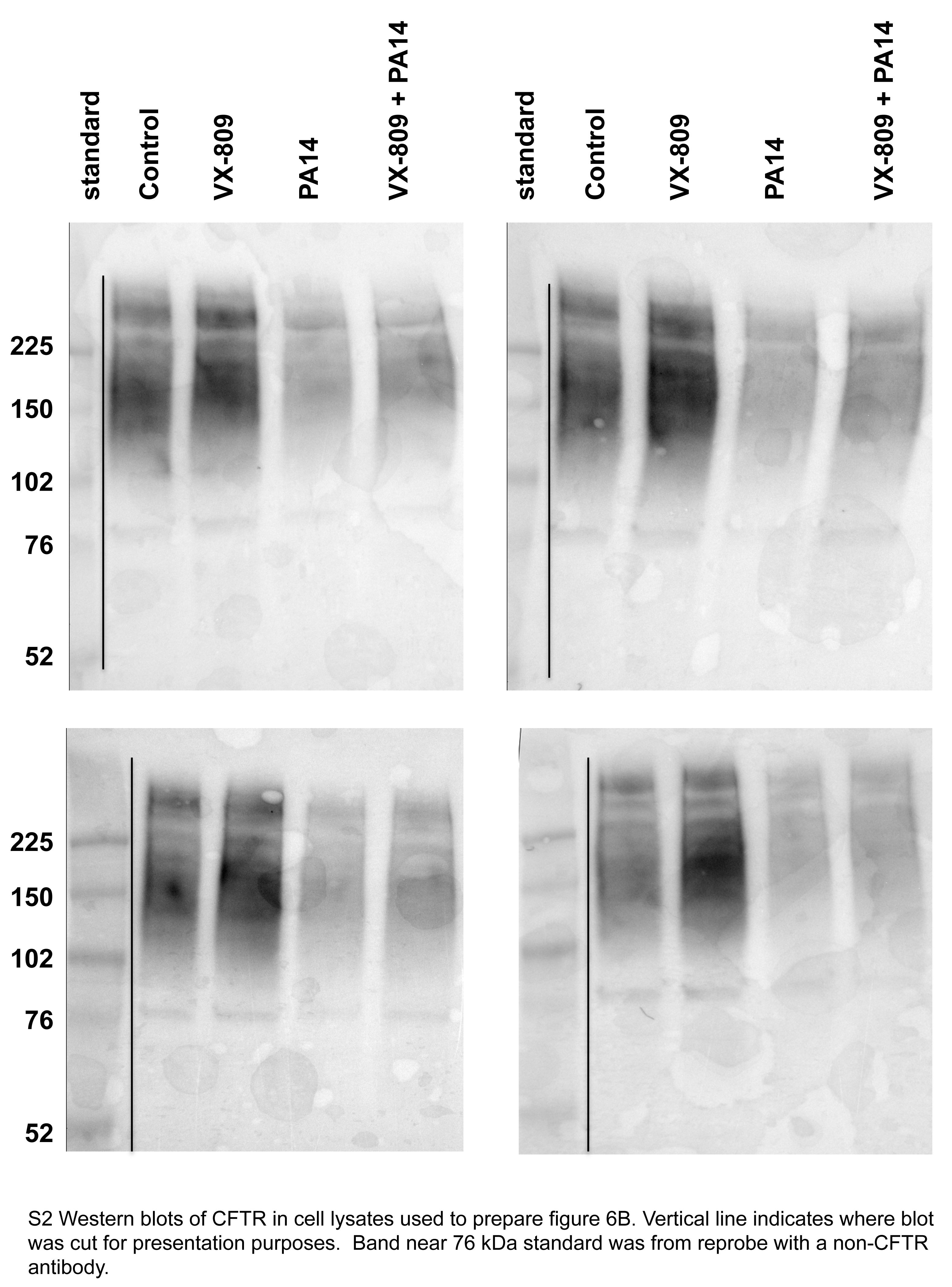

Supplement: S2 Fig — Vertical line indicates where blot was cut for presentation purposes. Band near 76 kDa standard was from reprobe with a non-CFTR antibody. (TIF) [file pone.0127742.s002.tif]

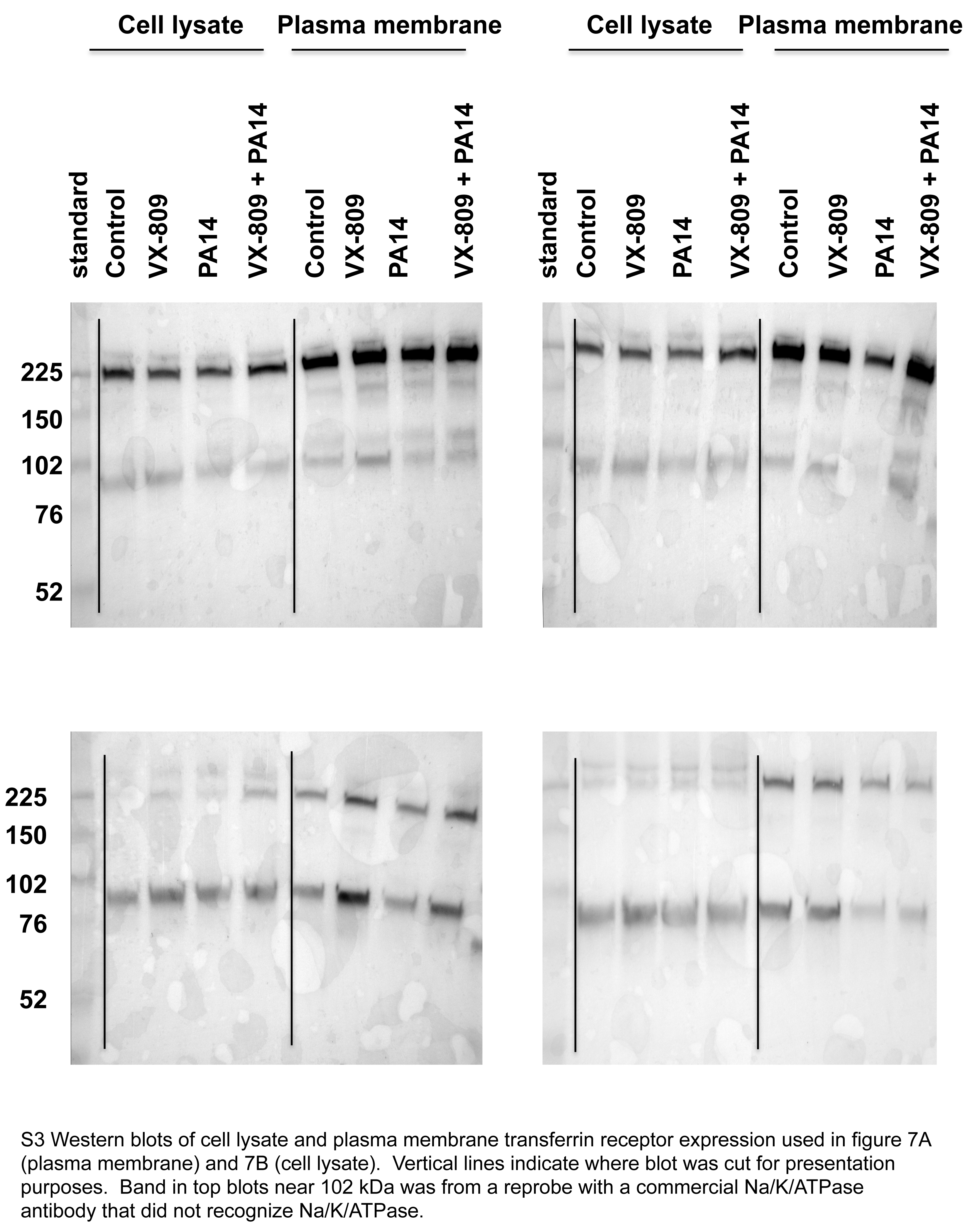

Supplement: S3 Fig — Vertical lines indicate where blot was cut for presentation purposes. Band in top blots near 102 kDa was from a reprobe with a commercial Na/K/ATPase antibody that did not recognize Na/K/ATPase. (TIF) [file pone.0127742.s003.tif]

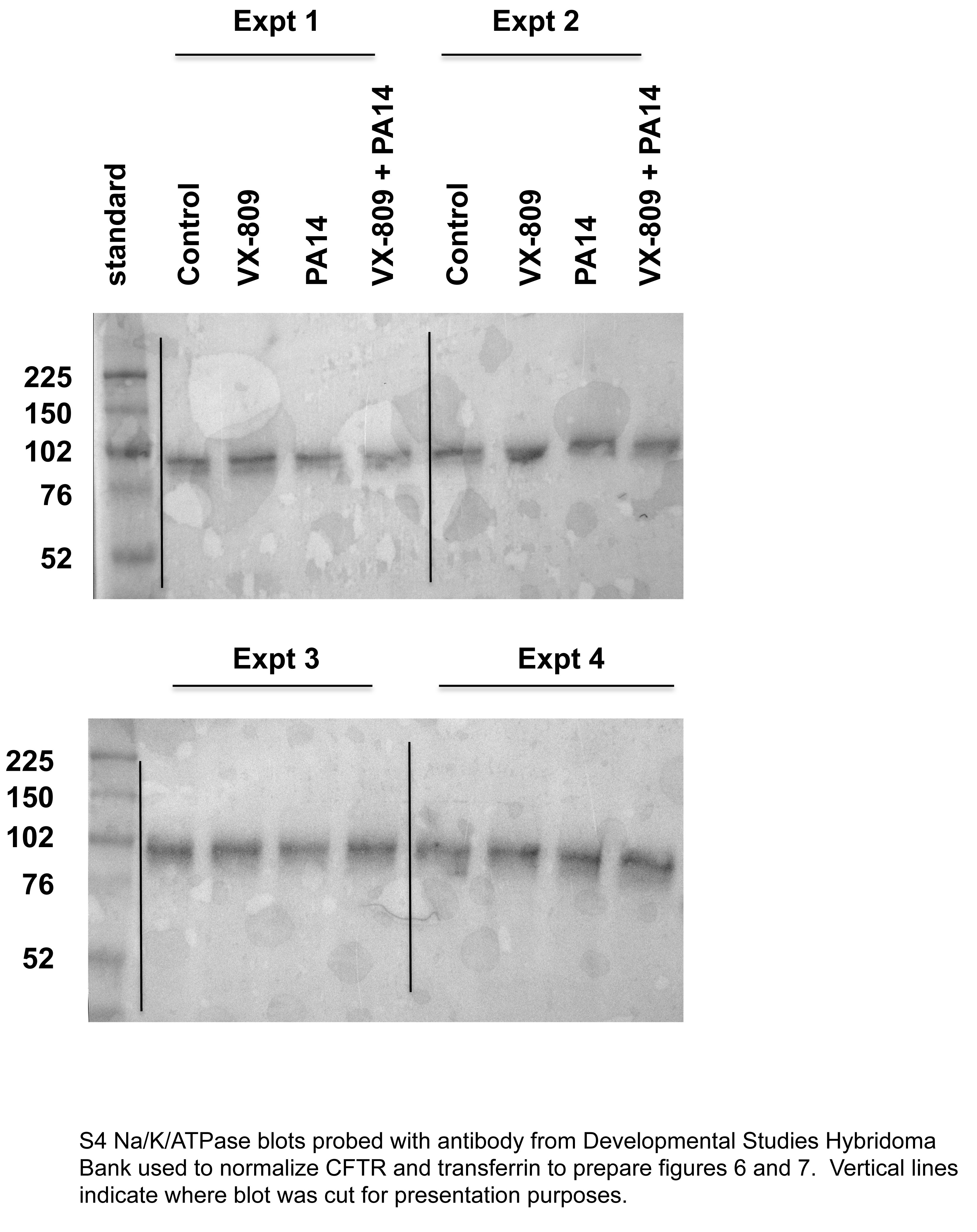

Supplement: S4 Fig — Vertical lines indicate where blot was cut for presentation purposes. (TIF) [file pone.0127742.s004.tif]
